# Automated threshold detection for auditory brainstem responses: comparison with visual estimation in a stem cell transplantation study

**DOI:** 10.1186/1471-2202-10-104

**Published:** 2009-08-26

**Authors:** Sofie Bogaerts, John D Clements, Jeremy M Sullivan, Sharon Oleskevich

**Affiliations:** 1 Neuroscience Research Program, Garvan Institute of Medical Research, Sydney, 2010, Australia; 2 Faculty of Medicine, University of New South Wales, Sydney, Australia

## Abstract

**Background:**

Auditory brainstem responses (ABRs) are used to study auditory acuity in animal-based medical research. ABRs are evoked by acoustic stimuli, and consist of an electrical signal resulting from summated activity in the auditory nerve and brainstem nuclei. ABR analysis determines the sound intensity at which a neural response first appears (hearing threshold). Traditionally, threshold has been assessed by visual estimation of a series of ABRs evoked by different sound intensities. Here we develop an automated threshold detection method that eliminates the variability and subjectivity associated with visual estimation.

**Results:**

The automated method is a robust computational procedure that detects the sound level at which the peak amplitude of the evoked ABR signal first exceeds four times the standard deviation of the baseline noise. Implementation of the procedure was achieved by evoking ABRs in response to click and tone stimuli, under normal and experimental conditions (adult stem cell transplantation into cochlea). Automated detection revealed that the threshold shift from pre- to post-surgery hearing levels was similar in mice receiving stem cell transplantation or sham injection for click and tone stimuli. Visual estimation by independent observers corroborated these results but revealed variability in ABR threshold shifts and significance levels for stem cell-transplanted and sham-injected animals.

**Conclusion:**

In summary, the automated detection method avoids the subjectivity of visual analysis and offers a rapid, easily accessible http://axograph.com/source/abr.html approach to measure hearing threshold levels in auditory brainstem response.

## Background

The auditory brainstem response (ABR) is a voltage response evoked by acoustic stimuli as sound is processed along the auditory pathway. It consists of electrical signals resulting from the sum of sound-evoked activity along the auditory nerve and brainstem nuclei. ABR analysis determines the sound intensity at which a neural response first appears (hearing threshold) [1]. Previous studies in rats in mice have shown that ABR thresholds do not indicate absolute behavioural hearing thresholds [2,3]. However, ABR audiometry has been used extensively in animal hearing research for examining gene therapy [4-10], cell-replacement therapies [11-14], and noise-induced hearing loss [15-20].

The ABR offers an objective measurement of auditory signal processing. The objectivity is diminished by conventional visual inspection of the ABR threshold level. Subjectivity and variability are introduced when the investigator has to decide when a complex, multi-component response first becomes distinguishable from background noise [21]. Methodologies have been developed to address the subjective component of threshold detection by including criteria about the shape, pattern, or absolute amplitude of the response, yet these still require a visual decision about the presence of a signal. Eliminating subjectivity in auditory threshold determination would improve the sensitivity and reliability of this important audiometric technique.

While visual estimation remains the conventional technique for ABR threshold detection, a need for automated statistical methods for detection is highly recognised. Several methods have been developed based on the techniques of F_sp _analysis [22-25], cross correlation [26-28] and feature vectors [29-32]. F_sp _analysis requires calculation of a variance ratio in the ABR waveform followed by application of the F-statistic to this ratio. Cross correlation measures the degree of similarity between a sliding template and an averaged waveform. Feature vectors quantify selected components of the response's time course. F_sp _has been incorporated into available software (Compumedics Ltd) yet the other methods lack comparable implementation.

Here we develop a simple, fully automated auditory threshold detection method to address the subjectivity and variability associated with visual estimation of ABRs. This method is based on the signal-to-noise ratio and the software has been made readily available [33]. The algorithm is calibrated by comparison with visual estimation, implemented via investigation of stem cell transplantation, and compared against variability obtained with visual estimation.

## Results and discussion

### Algorithm

**Figure 1:**
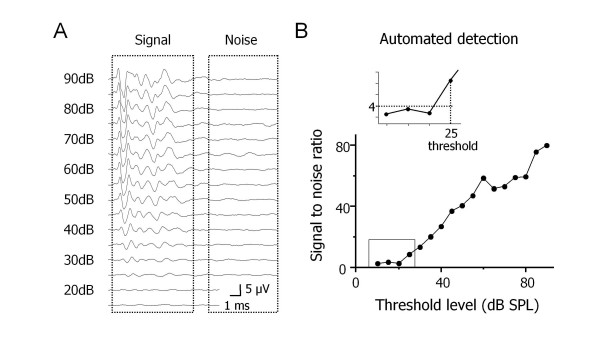
**Computational algorithm for ABR threshold detection**. **A**. Series of auditory brainstem responses (ABRs) evoked by click stimuli delivered at decreasing intensities. The automated threshold detection method compares the peak amplitude of the signal versus the standard deviation of baseline noise (boxed areas). **B**. Plot of the signal-to-noise ratio is used to calculate the hearing threshold level when peak amplitude is four times the standard deviation of the baseline noise to ensure few false positives (inset).

The automated method is based on the ratio between the observed peak amplitude of the evoked ABR signal and the standard deviation (SD) of the baseline noise. The peak was taken as the maximum absolute amplitude of the averaged evoked ABR signal in a time window encompassing the ABR signal (Figure 1). This peak amplitude represents the true peak amplitude plus a contribution from the background noise. The SD of the noise was calculated in a time window clearly following termination of the ABR signal. These calculations were repeated for each member of the family of voltage responses recorded at different sound intensity levels. The median value of the SDs was taken as the best available estimate of the true noise SD. The sound intensity level was deemed to have reached threshold when the peak ABR amplitude was four times the noise SD (Figure 1B). This signal-to-noise ratio was chosen after investigating other values (SD = 3 or 5) as it provides sensitive signal detection while maintaining a low probability of a false positive.

The automated detection method is based on a statistical foundation. Assuming a normal distribution of baseline noise data, 99.98% of noise values will lie within ± 4 SD of the mean. Any amplitude values occurring outside this range allow rejection of the null hypothesis (no ABR signal) at the p < 0.0002 level. This confidence level is valid when a single data point in the ABR signal is examined. If we search for a peak amplitude across a time window containing 100 data points, then a peak signal > 4 SD is significantly different from baseline noise at the p < 0.02 level, thus confirming the presence of an ABR signal and providing a statistically reliable estimate of the hearing threshold level.

An automated detection method based on the signal-to-noise ratio has previously been reported [22,23,25]. This method requires calculation of a parameter, F_sp_, which is the variance of the amplitude values across an specified time window of the averaged response (VAR(S)), divided by the variance of the amplitude of a single time point across several hundred sweeps (VAR(SP)). This deterministic approach assumes that the evoked ABR voltage waveform is constant from trial to trial yet neural population signals typically fluctuate in amplitude from trial to trial due to changes in the number of neurons contributing to the response. Such ABR amplitude fluctuations will cause VAR(SP) to be systematically overestimated. In contrast, assumption of biological variability has been included in our algorithm.

This relatively simple and intuitive threshold detection method was developed as a plug-in module for AxoGraph X, a data analysis application (AxoGraph Scientific). The automated analysis module, with source code, is freely available with an application license [33]. The module imports a graph containing the family of averaged voltage responses recorded at different sound intensity levels, then automatically outputs a plot of the signal-to-noise ratio versus sound intensity, with the ABR threshold level indicated.

### Automated method detects accurate and consistent hearing threshold levels

**Figure 2:**
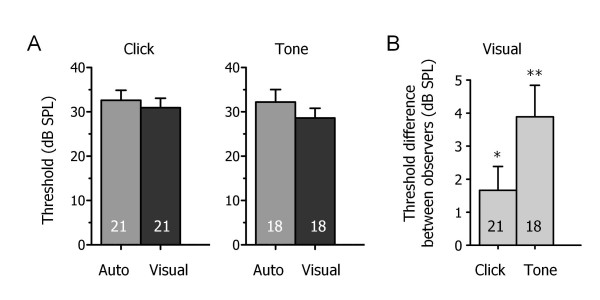
**Automated method detects accurate and consistent hearing threshold levels**. **A**. Summary data showing that automated detection with a signal-to-noise ratio value of four (SD ± 4) predicts equivalent and consistent threshold levels in comparison to visual estimation for click and tone (16 kHz) stimuli. **B**. The variability associated with visual estimation is evident as the absolute difference in threshold values between two observers was statistically different than zero for click (* p < 0.05) and tone stimuli (**p < 0.001). Values expressed as mean ± SEM for all graphs with number of animals indicated within columns.

To test the automated detection method, ABRs were evoked by acoustic stimuli (click and tone) and measured with subdermal electrodes in normal-hearing mice (Figure 1, 2). The ABRs consisted of five major wave components as reported previously [34-36]. In mice, the waves likely correspond to peripheral signal processing (wave I; auditory nerve) and central signal processing (waves II-V; cochlear nucleus, superior olivary complex, lateral lemniscus, inferior colliculus, respectively). Acoustic stimuli were delivered in descending intensities and hearing thresholds were detected by computerised automated detection and compared to visual estimations of hearing thresholds. For automated detection, peak signal and background noise measurements were made in time windows of 0.5–8 ms and 12–20 ms, respectively. A plot of signal-to-noise ratio versus sound intensity level was generated, and the ABR threshold level was indicated (see inset: Figure 1B). For visual estimation, two experienced observers (auditory clinician, neuroscientist) noted the lowest sound intensity that evoked an ABR.

For click and tone stimuli, automated detection produced a similar threshold value as visual estimation for the same series of ABR traces in normal-hearing mice (Figure 2). Summary data indicated that automated detection provided a mean threshold value of 33 ± 2 dB (n = 21) for click stimuli that was not significantly different than the mean threshold value predicted by visual estimation (31 ± 2 dB; n = 21; Figure 2A). This was also consistent in response to tone stimuli where automated detection showed a mean threshold value of 32 ± 3 dB (n = 18) while visual estimation predicted a mean threshold value of 29 ± 2 dB; n = 18; Figure 2A). Individual data revealed that automated detection produced identical estimates of ABR threshold compared to visual estimation in 48% of mice (n = 10/21) for click stimuli and in 50% of mice (n = 9/18) for tone stimuli. In the non-identical data, the threshold estimates for automated detection differed from visual estimation by a mean of 6 ± 0.6 dBs (n = 11) for click stimuli and by 9 ± 3 dBs (n = 9) for tone stimuli.

Two independent observers estimated significantly different threshold levels for the same series of ABRs (Figure 2B). The absolute difference in threshold values between observers was statistically different than zero for click stimuli (1.7 ± 0.7 dB; n = 21; p < 0.05) and for tone stimuli (3.9 ± 0.9 dB; n = 18; p < 0.001). Together, the results confirm that a signal-to-noise ratio value of four (SD ± 4) predicts equivalent and consistent threshold levels in comparison to visual estimation and that variability in threshold detection is associated with visual estimation.

### Automated detection method is used to investigate vestibular stem cell transplantation

**Figure 3:**
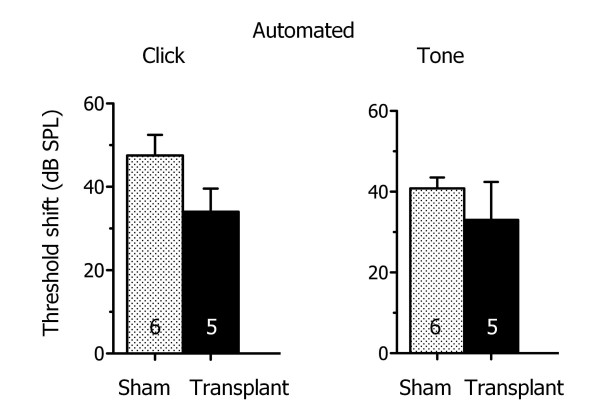
**Automated detection method is used to investigate vestibular stem cell transplantation**. The mean difference in ABRs before and after surgery (threshold shift) was not significantly different for mice transplanted with vestibular stem cells compared to sham-injected mice in response to click and tone stimuli.

The automated threshold algorithm was implemented to investigate the effect of adult stem cell transplantation into the cochlea on hearing threshold levels (Figure 3). Stem cell transplantation is rapidly gaining interest as a potential therapy for hearing loss (for review see [37,38]). Our recent findings show that transplantation of adult tongue stem cells into deafened mice results in a significantly smaller mean ABR threshold shift compared to sham injection [39]. Previous studies suggest that vestibular stem cells reside in the vestibular sensory epithelium, and are pre-programmed to differentiate into vestibular hair cells that share similarities with cochlear hair cells [40-42]. Here we use the automated threshold algorithm to determine ABR changes after adult vestibular cell transplantation. Acoustic deafening prior to transplantation, as performed in our earlier studies, was omitted to investigate the specific effects of stem cells on transplant surgery. Hearing thresholds were measured four weeks after transplantation of a cell suspension containing vestibular stem cells or sham injection of the vehicle (phosphate buffer) alone. Comparisons were made of the shift between pre- and post-surgery ABR threshold levels in the treated ear (Figure 3).

The mean ABR threshold shift between pre- and post-surgery hearing levels for mice transplanted with vestibular cells was 34 ± 6 dB (n = 5) in response to click stimuli. This threshold shift was not significantly different than for mice receiving a sham injection (48 ± 5 dB; n = 6). For pure tone stimuli, the mean ABR threshold shift was also not significantly different for stem cell-transplanted (33 ± 9 dB; n = 5) versus sham-injected animals (41 ± 3 dB; n = 6; Figure 3B).

### Automated detection avoids variability associated with visual estimation

**Figure 4:**
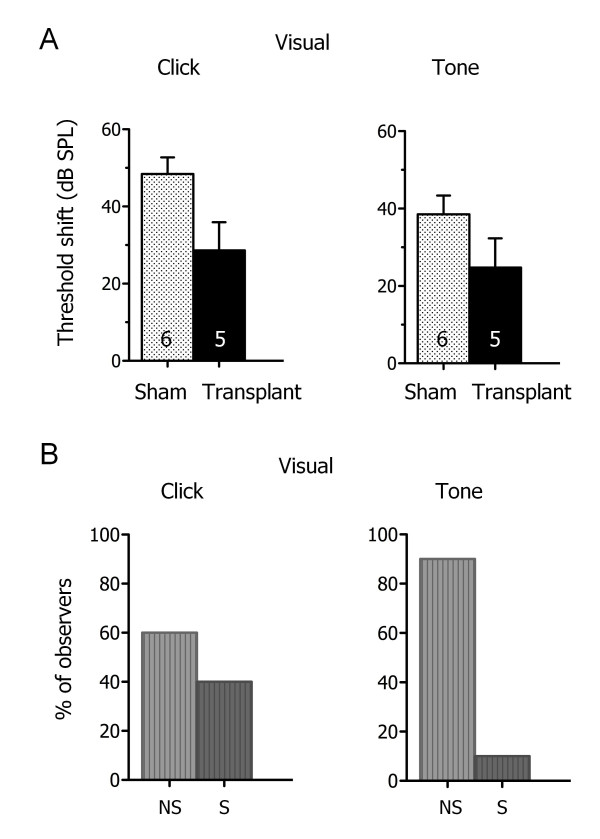
**Automated detection avoids variability associated with visual estimation**. **A**. Mean ABR threshold shift for stem cell-transplanted and sham-injected mice in response to click and tone stimuli as estimated by visual inspection. The mean value for each animal was calculated from the data of ten independent observers. Comparisons of the two cohorts reveal similar results as for data analyzed by automated detection (see Figure 3). **B**. Examination of the data for individual observers shows that most observers did not detect a significant difference between stem cell-transplanted and sham-injected mice (NS) while some observers noted a significant difference in threshold shifts (S; p < 0.05). This variability in significance levels differed between click and tone stimuli.

To assess the subjectivity and variability associated with visual estimation of ABR threshold levels (Figure 4), ten independent observers assessed the threshold levels on the ABRs previously analyzed by automated detection (see Figure 3). The observers were blind to the experimental conditions, and included experienced auditory clinicians and neuroscientists. Visual estimation corroborated the results provided by automated detection, showing that threshold shifts between stem cell-transplanted and sham-injected mice were not significantly different in response to click and tone stimuli (Figure 4A). However examination of the data for individual observers shows that while most observers did not detect a significant difference in threshold shifts, some observers noted a significant difference (p < 0.05) between stem cell-transplanted and sham-injected mice (Figure 4B). This variability in significance levels differed for click and tone stimuli. Thus the automated detection method predicts similar mean threshold values as visual estimation, in agreement with the results obtained for normal-hearing mice (Figure 2A), and avoids the variability in ABR threshold shifts and significance levels associated with visual estimation.

## Conclusion

A simple, fully automated auditory threshold detection method was developed to address the subjectivity and variability associated with visual estimation of ABRs. The threshold values provided by automated detection were similar to those provided by visual estimation, indicating the automated method predicts valid threshold levels. The automated detection method was implemented in experimental conditions and revealed no difference between stem cell-transplanted and sham-injected mice. Visual estimation by independent observers corroborated these results but revealed variability in ABR threshold shifts and significance levels for stem cell-transplanted and sham-injected animals. In summary, the automated detection method developed here offers an accessible, accurate, and reproducible approach for measuring hearing threshold levels in auditory brainstem responses.

## Methods

### Auditory Brainstem Responses (ABRs)

Auditory function was assessed by measuring ABR thresholds in CBA/CaH mice aged 4 to 6 postnatal weeks (n = 41) as previously described [43]. ABR thresholds were recorded in two groups of animals: normal hearing mice (n = 30) and mice receiving a unilateral stem cell transplantation or sham injection into the left cochlea (n = 11). Briefly, mice were anaesthetized with ketamine (100 mg/kg) and xylazine (20 mg/kg), and ABRs were recorded differentially between subdermal platinum electrodes placed at the vertex and lateral to the left cheek with an electrode at the lower back serving as ground. Clicks (1 ms duration, 100 ms interstimulus interval) and tone pips (16 kHz; 1 ms rise/fall; 3 ms duration, 90 ms interstimulus interval) were delivered via an electrostatic insert speaker and ABRs were obtained by reducing the intensity in 5 dB steps beginning at 90 dB (Tucker Davis Technologies). The ABR signal was obtained by time locked averaging with a minimum of 512 averages. ABRs were band passed filtered above 300 Hz and below 1500 Hz. No stimulus artifact was observed. With ideal recording conditions, a baseline noise of 100–200 μV was achieved. ABRs were recorded with BioSig software (Tucker Davis Technologies) and converted to TIFF files for visual estimation in printed format or to ASCII files for automated computer analysis.

All experiments were performed with the approval of the Garvan Institute and St Vincent's Hospital Animal Ethics Committee, in accordance with the Australian Code of Practice for the Care and Use of Animals for Scientific Purposes (National Health and Medical Research Council, 2004).

### Stem cell transplantation

For vestibular primary cell culture, male and female CBA/CaH mice (n = 5; aged 10–15 postnatal days) were anesthetized with CO_2 _and decapitated. Inner ears were placed in chilled Dulbecco's modified Eagle medium (D-MEM) containing 9.6 mg/ml HEPES, and the utricular maculae and ampullary cristae carefully dissected. The outer margins of the sensory epithelia were then trimmed away and the tissues processed according to a method adapted from that of Oshima et al. [42]. Tissues were incubated in 0.5 mg/ml thermolysin (Sigma) in D-MEM for 30 min at 37°C and then in 0.125% trypsin in Hank's Balanced Salt Solution for 20 min at 37°C. Tissues were washed in Advanced D-MEM/F-12 medium containing 20 mM glutamine and 10% fetal bovine serum and gently triturated. Dissociated cells were centrifuged at 400 × g and resuspended in 10 ml Advanced D-MEM/F-12 medium containing 20 mM glutamine, B-27 supplement minus vitamin A, N2 supplement, 20 ng/ml EGF, 20 ng/ml bFGF (both Millipore), 100 U/ml penicillin G and 100 μg/ml streptomycin. The cell suspension was then poured through a 70 μm cell strainer (BD Falcon) into plastic tissue culture dishes (BD Falcon) and cultured at 37°C with 5% CO_2_. Cells were collected after seven days *in vitro *for transplantation experiments after dissociation with TrypLE Express. All reagents from Invitrogen unless otherwise stated.

For transplant surgery, cochleostomies were performed on CBA/CaH mice aged 4 to 6 postnatal weeks (n = 11) as previously described [43]. Briefly, minimal trauma surgery was performed on mice anaesthetised with 75 mg/kg ketamine and 15 mg/kg xylazine. A minimally invasive procedure was initiated by micro drilling through the bulla to access the inner ear and perform a lateral wall cochleostomy in the basal turn of the cochlea. Transplantations were made using a glass capillary needle (tip diameter of 100 μm) inserted into the cochleostomy. For stem cell injections (n = 6), one microliter of stem cells suspended in phosphate buffer was injected over one minute to deliver 2000–4000 cells. For sham injections (n = 5), identical techniques were followed except that phosphate buffer was substituted for the stem cell solution.

Statistics are quoted as mean ± standard error of the mean (SEM). Significant differences in mean threshold values were determined using a two-tailed unpaired t-test (Prism, GraphPad).

## Authors' contributions

SB recorded auditory brainstem responses, performed transplantation surgery and data analysis, and aided in manuscript preparation. JDC developed the computer software for automated detection and aided in manuscript preparation. JMS prepared vestibular stem cells for transplantation and aided in manuscript preparation. SO participated in design of the study, performed the statistical analysis, and prepared the manuscript. All authors read and approved the final manuscript.

## Authors' information

The authors SB, JMS, and SO have no competing interests; JDC is a software programmer at AxoGraph Scientific.
